# Commentary: The effects of creatine supplementation on cognitive function in adults: a systematic review and meta-analysis

**DOI:** 10.3389/fnut.2026.1716285

**Published:** 2026-04-10

**Authors:** Tom Citherlet

**Affiliations:** University of Lausanne, Institute of Sports Sciences, Synathlon, Lausanne, Switzerland

**Keywords:** cognition, creatine, double counting, meta-analysis, systematic review

## Introduction

The recent article by Xu et al. (1) concludes that creatine supplementation has positive effects on cognitive performance in adults. The effort to synthesize the available evidence on this important question is greatly appreciated. However, certain aspects of the statistical approach have introduced a unit-of-analysis error.

## Double-counting of non-independent outcomes

Several included trials report multiple correlated cognitive outcomes from the same participants, yet these outcomes are treated as independent effect sizes. For example, in Figure 8 (memory), Alves (2013a, 2013b) each provide at least seven memory subtests, McMorris (2006) four, McMorris (2007b) four, and Pires (2020) four. Consequently, the number of observations in the pooled analysis exceeds the number of unique randomized participants. This “double-counting” violates the assumption of independent observations and is known to artificially inflate precision and statistical power (2).

## Evidence from previous analyses

A closely related issue occurred in the meta-analysis by Prokopidis et al. (3) on creatine's effects on memory. In a subsequent letter, Eckert and Pascher (4) showed that including multiple non-independent outcomes from the same participants leads to statistical distortions and increases the risk of false-positive findings. When Prokopidis and colleagues re-analyzed their data using an appropriate method, the overall effect of creatine on memory reported in their 2023 study was no longer significant, except in older adults.

## EFSA panel's critique of the meta-analysis

The European Food Safety Authority (EFSA) (5) highlighted the same concern in its 2024 scientific opinion on creatine and cognition, noting that pooling non-independent cognitive test results in the meta-analysis by Xu et al. (1) inflated sample sizes. Consequently, the EFSA determined that no conclusions could be drawn based on that data. As this point is not readily visible to readers of the meta-analysis, linking it to a commentary may provide helpful context.

## Discussion

To properly resolve this unit-of-analysis error, a re-analysis of the data using appropriate meta-analytic approaches is recommended, such as applying multilevel models to account for nested data or averaging multiple non-independent outcomes within individual studies prior to analysis. These remarks are offered constructively, with the hope that a careful re-analysis could further strengthen this important contribution to the literature and clarify whether the apparent cognitive benefits of creatine supplementation are supported by the evidence.
